# Testing hypotheses for the function of the carnivoran baculum using finite-element analysis

**DOI:** 10.1098/rspb.2018.1473

**Published:** 2018-09-19

**Authors:** Charlotte A. Brassey, James D. Gardiner, Andrew C. Kitchener

**Affiliations:** 1 School of Science and the Environment, Manchester Metropolitan University, Manchester M1 5GD, UK; 2 Institute of Ageing and Chronic Disease, University of Liverpool, Liverpool L7 8TX, UK; 3 Department of Natural Sciences, National Museums Scotland, Chambers Street, Edinburgh EH1 1JF, UK

**Keywords:** baculum, biomechanics, copulation, finite-element analysis, genitalia, intromission

## Abstract

The baculum (os penis) is a mineralized bone within the glans of the mammalian penis and is one of the most morphologically diverse structures in the mammal skeleton. Recent experimental work provides compelling evidence for sexual selection shaping the baculum, yet the functional mechanism by which this occurs remains unknown. Previous studies have tested biomechanical hypotheses for the role of the baculum based on simple metrics such as length and diameter, ignoring the wealth of additional shape complexity present. For the first time, to our knowledge, we apply a computational simulation approach (finite-element analysis; FEA) to quantify the three-dimensional biomechanical performance of carnivoran bacula (*n* = 74) based upon high-resolution micro-computed tomography scans. We find a marginally significant positive correlation between sexual size dimorphism and baculum stress under compressive loading, counter to the ‘vaginal friction’ hypothesis of bacula becoming more robust to overcome resistance during initial intromission. However, a highly significant negative relationship exists between intromission duration and baculum stress under dorsoventral bending. Furthermore, additional FEA simulations confirm that the presence of a ventral groove would reduce deformation of the urethra. We take this as evidence in support of the ‘prolonged intromission’ hypothesis, suggesting the carnivoran baculum has evolved in response to pressures on the duration of copulation and protection of the urethra.

## 1. Introduction

The baculum (or os penis) is a mineralized bone located within the glans of the mammalian penis. The baculum is considered one of the most morphologically diverse bones within the mammalian skeleton [1,2] and its presence has been documented across nine modern Orders: Afrosoricida, Carnivora, Chiroptera, Dermoptera, Erinaceomorpha, Lagomorpha, Primates, Rodentia and Soricomorpha [3,4]. Yet, the evolutionary history of the baculum has only recently been resolved following the application of modern phylogenetic techniques [4,5]. Most strikingly, the structure is now suspected to have arisen independently on at least nine occasions over the course of placental mammal evolution [4]. The common ancestor of carnivorans possessed a baculum, for example, which was lost in two independent lineages (hyaenas and binturong). By contrast, the baculum was gained at least twice in Primates (in Strepsirhini, and separately in Simiiformes), with the latter followed by three subsequent losses [4].

While historically it has been argued that baculum variability may represent a pleiotropic by-product of phyletic divergence [6], a recent body of experimental work in mice has provided compelling evidence for the baculum being subject to direct sexual selection [7,8]. Yet despite several hypotheses being put forward, the adaptive function of the baculum during copulation remains unclear and may vary across Orders [9]. This lack of consensus is particularly noteworthy given the relative ubiquity of the structure [4] and the fascination it holds for natural historians.

A potential role for the baculum in female stimulation has been suggested by some authors. The ‘induced ovulation’ hypothesis [10] posits that the stiffness and/or tip shape conferred by the baculum during copulation may stimulate the female and trigger the release of ova in taxa that do not otherwise ovulate spontaneously. Several issues have been raised with this theory, however [11], not least that groups characterized by very reduced/absent bacula (artiodactlys, rabbits, cats) also experience induced ovulation. In addition, a lack of species-specific data on mammalian ovulation mechanisms complicates potential comparative analyses.

Alternatively, the ‘vaginal friction’ hypothesis proposes that the baculum may impart additional rigidity to the penis to achieve intromission [12]. In this instance, sexual size dimorphism (SSD) is taken as a proxy for friction, assuming that males copulating with relatively smaller females would struggle to achieve intromission owing to their comparatively smaller vaginal tract (although see Discussion). While this theory has gained some support [2], a broader analysis has failed to find a correlation between baculum size (measured as length) and SSD across carnivorans [11].

Finally, the ‘prolonged intromission’ hypothesis suggests that the baculum may prevent the ventrally located urethra from becoming occluded during copulation, ensuring semen is effectively delivered [13]. The baculum is, therefore, hypothesized to increase in relative size in taxa engaging in prolonged intromission, particularly those adopting copulatory ties, such as canids. Previous studies have subdivided taxa into ‘short’ and ‘long’ intromission periods at 3 min (with seemingly little biological justification), and have found support for this hypothesis among primates and carnivorans [5,14,15]. However, when intromission length is treated as a continuous variable, the results become more mixed. No relationship between normalized baculum length and intromission duration was identified in a small sample of carnivorans [11], while a significant correlation has been found in a larger combined dataset of carnivorans and primates [16] (although see later discussion re: a lack of homology). In a particularly noteworthy study, Herdina *et al*. [17] found experimental evidence for the baculum straightening and protecting the urethra in the erect cadaveric genitals of bats.

While the above hypotheses rest upon an assumption of a ‘mechanical’ function for the baculum, it is conspicuous that previous studies have relied upon simple linear metrics (length, diameter) to *infer* biomechanical performance. Given the well-documented diversity in baculum morphology, studies risk overlooking the potentially important interaction between size *and* shape when testing functional hypotheses. By approximating the baculum to a simple rod, features such as shaft curvature, tip ornamentation and urethral grooves have been ignored in past quantitative analyses.

For the first time, to our knowledge, we apply a three-dimensional computational biomechanical simulation technique to quantify the loading behaviour of a broad sample of carnivoran bacula. We generate high-resolution three-dimensional models of the carnivoran baculum, using micro-computed tomography (μCT), and subject the models to simulated bending and compressive loads using finite-element analysis (FEA). FEA is an engineering technique that estimates deformation, strain and stress within an object when subjected to loads [18]. A virtual mesh of the object is generated, comprising a large number of simple ‘elements’. The mesh is constrained at selected nodes, a load applied to other nodes, and a series of partial differential equations solved numerically in order to calculate displacement at each node of the mesh. Rather than modelling the baculum as a simple beam, the application of FEA allows for the entire geometry of the object to be incorporated. This is essential when faced with a comparative sample characterized by high morphological disparity in aspect ratio, cross-sectional geometry and curvature [19], such as is present in mammalian bacula.

Specifically, we apply FEA to calculate a metric of relative ‘robustness’ of the baculum under a given loading scenario. We subsequently test two hypotheses explicitly pertaining to the biomechanical function of the baculum.

(*H*_1_) *The ‘vaginal friction’ hypothesis*: taxa characterized by higher SSD will possess relatively more robust bacula when subject to compressive loading, similar to that expected to occur during initial vaginal penetration.

(*H*_2_) *The ‘prolonged intromission’ hypothesis:* taxa characterized by longer periods of intromission will possess relatively more robust bacula when subject to bending loads, to limit occlusion of the urethra and aid delivery of sperm.

In addition, we include FEA models of simplified baculum morphs to simulate the effect of incorporating a urethral groove on predicted stress values. In doing so, we examine the potential role of the urethral groove in protecting the urethra against deformation during loading.

Here, we choose to focus on carnivorans as a recent analysis has found the baculum to be homologous within the group [4]. Previously, a *combined* dataset of primate plus carnivoran bacula has been used to test functional hypotheses for the baculum. However, in light of recent evidence for the independent origin of the baculum in several orders of mammal, it is prudent to restrict our hypothesis testing to taxa for which we are confident the baculum is a homologous structure. Furthermore, SSD within the Carnivora ranges from negligible (herpestids) to extreme (mustelids, ursids), intromission varies from very short (less than 1 min, felids) to very long (greater than 3 h, fossa), and there is extreme diversity in baculum shape and size. We emphasize that the present study does not replicate any *known* loading regime of carnivore genitalia, as these data simply do not exist. The inherently ‘hidden’ nature of intromission within internally fertilizing species has precluded any kinematic or kinetic analysis of copulation in this group. The direction and magnitude of *in vivo* loads are unknown, although the occurrence of healed fracture calluses implies bacula are loaded to failure on occasion [20].

Rather, we seek to quantify the comparative robustness of bones under hypothetical loading scenarios. By taking this novel ‘whole bone’ approach to hypothesis testing, this study is an important contribution to our understanding of the functionality of this common component of mammalian anatomy. More broadly, the field of reproductive biomechanics has lagged behind that of locomotor, feeding and respiratory biomechanics, despite its obvious evolutionary importance [21]. We hope the present application of computational simulation techniques to animal genitalia represents an important step towards a rigorous biomechanical approach to reproductive anatomy.

## 2. Methods

### (a) Computed tomography

Carnivoran bacula (*n* = 74) were sourced from the osteological collections of the National Museums Scotland, Edinburgh (NMS) and the Natural History Museum, London (NHM) (see the electronic supplementary material, table S1). Specimens with associated life-history data (body mass, age, location of collection) were preferred when available. Specimens were excluded if they showed signs of pathology, post-mortem damage or sub-adult status.

NMS specimens were μCT scanned at Manchester X-ray Imaging Facility in a 320/225 Nikon XTEK custom bay. NHM specimens were μCT scanned at the museum's Imaging and Analysis Centre using a Nikon Metrology HMXST 225 scanner (see the electronic supplementary material, table S2). Specimens were digitized within a single scan volume, with the exception of the pinnipeds and polar bear (*Ursus maritimus*). For larger specimens, multiple scans were conducted down the specimen long axis and stitched together in post-processing.

Carnivorans vary in the occurrence of a cartilaginous tip at the distal end of the baculum (comprising up to 10% of total length in canids [22], yet absent in mustelids). Cartilage is not preserved in osteological museum specimens and is not included in the present analysis. Unfortunately, the degree to which ‘modelled’ bacula represent the morphology of bacula *in vivo* will therefore differ, and this error will contain a phylogenetic signal. Given the paucity of data pertaining to cartilaginous tips, there is currently no mechanism by which to correct the models. A future focus on digitizing cadaveric ‘wet’ specimens would be beneficial in this regard.

In this study, all specimens were scanned as excised dried bacula, with the exception of felids. Cat bacula are small in absolute size and rarely found within natural history collections. Whole intact cat penes were sourced from the NMS, fixed in 70% ethanol and bacula scanned *in situ* within the glans tissue. Dried bacula were mounted vertically in foam during scanning. Fixed cat penes were wrapped in gauze and secured vertically inside plastic tubing to minimize movement artefacts during scanning. Scans were reconstructed in Nikon CTPro, exported as 32-bit .vol files and subsequently read into Avizo 9.4 (FEI Visualization Sciences Group, Hillsboro, Oregon). When multiple specimens were present within a single scan, a rectangular region-of-interest was used to crop individual bacula, which were then saved as separate .raw files. When sequential scans were required to capture the length of larger bacula, the individual scans were translated along the long axis using the ‘Transform’ function and stitched together using the ‘Merge’ function. In some instances, low-density dried soft tissue was still attached to the outer surface of the baculum, or museum labels and string remained attached. These features were manually segmented from the scan using the ‘Edit Label Fields’ function, prior to subsequent export. Individual .raw files were converted to binary data in ImageJ by automated thresholding according to the histogram of raw CT greyscale values and exported as 8-bit data. Two taxa (*Melogale moschata* and *Lycalopex griseus*) were removed from the analysis at this stage. CT and visual inspection revealed highly porous ‘bubbly’ bone which was otherwise absent from very closely related taxa and was taken as evidence of pathology.

Principal axes were calculated using the ‘Moments’ function of the ImageJ plugin ‘BoneJ’ [23], and each specimen rotated such that the principal axes align with the image stack's *x-*, *y-* and *z-*axes. Image data were subsequently reimported into Avizo, and three-dimensional tetrahedral meshes generated using the ‘Meshing’ functionality, using ‘fast meshing’ and ‘optimize mesh’ options. Four-node linear tetrahedra were preferred for FEA as previous research has found such meshes perform well when compared with *ex vivo* experimental strain magnitudes [24]. Furthermore, FEA strain values have been found to converge when element numbers exceed 200 000 [24] to 800 000 [19] elements. Here, meshes comprised on average 2 million elements (mean 2 150 467, range 288 326–5 527 826). Meshes were exported from Avizo in the proprietary Abaqus FEA solver (Dassault Systems, Paris) file format.

### (b) Finite­-element analysis

**Figure 1.**
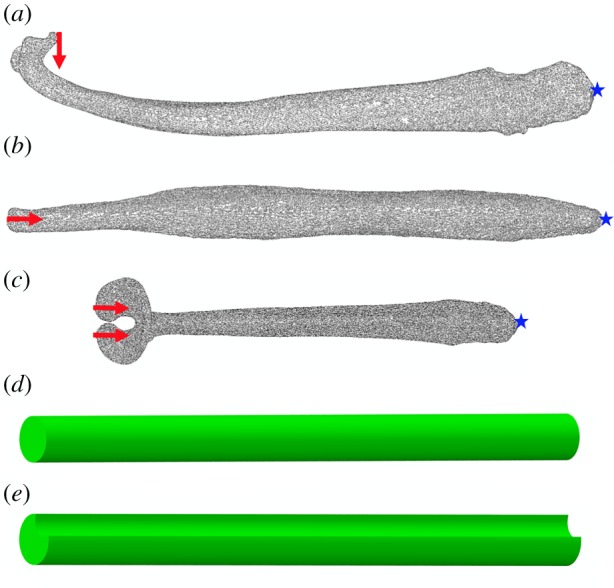
Generation of finite-element models. (*a–c*) Arrows indicate location and direction of applied loads on distal tip. Star indicates location of encastré boundary conditions. (*a*) Lateral view of *Mustela putorius* baculum under bending scenario. Loads are applied to the ‘anatomical’ tip of the baculum, as opposed to the most geometrically distal nodes of the model. (*b*) Dorsal view of *Canis aureus* baculum under compressive loading scenario. (*c*) Dorsal view of *Pteronura brasiliensis* baculum under compressive loading scenario. When bifurcation of the tip is present, an equal number of nodes on both sides are subject to loading. (*d*) Beam model without a groove. (*e*) Beam model with well-developed ventral groove (rotated to face dorsally for illustrative purposes). Note, model (*d*) and model (*e*) comprise the same total volume, by virtue of an increased radius in model (*e*). (Online version in colour.)

Within Abaqus, material properties were set to a Young's modulus of 5.3 GPa and Poissons ratio of 0.3. Young's modulus represents the ability of a material to withstand deformation. Poisson's ratio is calculated as the proportional decrease in unit width proportional to the increase in unit length when a material is elastically stretched. A Young's modulus of 5.3 GPa was derived from eight bacula sourced from intact (non-castrated) domesticated dogs subjected to mechanical 4-point bend testing [25]. As far as the authors are aware, these are the sole published material properties for the carnivoran baculum (see Discussion). Baculum models were simulated undergoing dorsoventral bending and compression. Under both scenarios, bacula were fixed at the base using encastré boundary conditions applied to 20 proximal nodes, such that the nodes were prevented from displacing or rotating (figure 1).

Concentrated force loads were applied to the tip of the baculum at 20 distal nodes. Nodes subject to loading were selected as being the most distal *anatomically* (i.e. closest to the urinary meatus), as opposed to *geometrically* (i.e. most positive location along the *z*-axis). This is particularly relevant in the case of bacula of the Mustelinae, which display extreme recurvature of the long axis, such that the apex of the curve is distal to the bone tip (figure 1*a*). When the distal end of the baculum terminated in one discrete ‘tip’, force was applied to this location (figure 1*b*). In instances of bifurcation in the distal baculum, 10 nodes per ‘tip’ were loaded (figure 1*c*). To simulate dorsoventral bending, loads were orientated in a ventral direction, whilst compression was simulated by loading directly down the *z*-axis (major axis) of the bone. Applied loads were set at 1% of male body mass, maintaining equivalency of loads across taxa (although see Discussion) and ensuring deformation remained within the linear elastic regime. When an associated body mass was available for specimens, this value was used. Otherwise, body mass values were sourced from the literature (see the electronic supplementary material, S1).

Models were solved on a Mac Pro (3.7 GHz Quad-Core Intel Xeon E5, 64GB 1866 MHz DDR3 RAM) and took an average of 6 min (range 30 s to 30 min). Values for Von Mises stress (*σ*_VM_) at each node were subsequently exported as an .rpt file for further analysis. Von Mises stress combines the three principal stresses into one equivalent stress and is a good indicator of material failure in ductile materials such as bone. Ductile failure would be predicted when *σ*_VM_ exceeds the yield strength for a given material. Here, we calculate mean values of *σ*_VM_ for each model as an overall metric of robustness under the two loading scenarios, as per previous studies [26,27]. The distribution of *σ*_VM_ across models was compared via visual inspection of three-dimensional stress maps.

### (c) Statistical analysis

We test for the presence of a phylogenetic signal in *σ*_VM_ using the ‘phylosig’ function of the ‘phytools’ package [28] of R to calculate Pagel's lambda (*λ*), based on a consensus carnivore phylogeny from the 10kTrees project [29]. *λ* values typically range between 0 and 1. As lambda approaches 1, traits follow a purely Brownian motion model of evolution, whilst *λ* = 0 suggests no correlation between species relative to the correlation expected under Brownian evolution. The maximum-likelihood estimates of the lambda model were compared to an assumption of *λ* = 0 using a likelihood ratio test following a Chi-squared distribution. A further four taxa were removed at this stage as they were not represented in the consensus phylogeny.

SSD was defined as log(male body mass/female body mass) [30] on the basis of literature values for body mass. When sex-specific mass data were not available, taxa were excluded from further analysis (two taxa of 70 total). One instance of intromission was defined as a single period of mounting by the male. In coati (*Nasua*), for example, males mount females for up to 60 min, during which 2–5 s bouts of thrusting are separated by 1–5 s pauses [31]. Here, we take 60 min as total intromission duration. By contrast, male large cats engage in short periods of mounting ranging from 3 s to 1 min, followed by dismounting and subsequent remounting, and may occur several times within an hour [32]. In this instance, we take the short period of mounting as representative of intromission duration. In canids, the duration of intromission is inclusive of the copulatory tie. If a range of values were reported, maximum recorded intromission duration for a given study was used. When intromission data were not available, taxa were excluded from further analysis (23 taxa of 70 total). Unlike previous studies [5,14], intromission duration was treated as a continuous variable, as opposed to subdividing taxa into ‘short’ and ‘long’ intromission groups at the 3 min mark.

Phylogenetically corrected phylogenetic generalized least squares (PGLS) linear regressions of *σ*_VM_ against SSD and intromission duration were conducted with the ‘gls’ function in R package ‘nlme’ [33], using the ‘corPagel’ function of the ‘ape’ package to simultaneously estimate both the regression model and *λ* of the residual error using maximum-likelihood. Regressions were conducted on log_10_-transformed data. Quadratic regressions were also fitted to the data in order to test for a curvilinear relationship between intromission duration and *σ*_VM_. In this instance, the second-order variable was generated by log_10_ transforming and centering intromission duration on the mean, prior to squaring centred intromission lengths. Centering the data was carried out to reduce multicollinearity between predictor variables [34]. Quadratic models were compared to linear models on the basis of Akaike information criterion (AIC) scores. PGLS analyses were also repeated by regressing baculum length against SSD and intromission duration, to compare results gained from three-dimensional FEA analysis to more traditional linear metrics used previously [5,11].

Simulations were also performed to quantify the level of type I error present within the PGLS regressions specific to the phylogeny. Type I error was estimated by simulating (*n* = 10 000) two random traits onto the phylogeny while incorporating estimated *λ*, and the proportion of iterations in which a correlation is detected ‘by chance’ quantified. A second set of simulations were conducted in which one trait evolved randomly whilst the second trait evolved with a known correlation to the first. Correlation levels were set at *β*_1_ = 0.75 and 0.90, as *per* Revell [35]. The power of the PGLS to detect a correlation was taken as the proportion of iterations in which a significant (*p* < 0.05) relationship was identified.

### (d) Urethral groove simulation

Hypothetical beam models were subject to FEA to simulate the effect of incorporating a urethral groove in the baculum. As a starting point, a solid beam of length 140 mm and circular cross-section of 5 mm radius was modelled in Abaqus. The same material properties were assigned to the beams as per the bacula above. One end surface was fixed under encastré boundary conditions, and the opposite end loaded with 9.8 N of force (equivalent to 1% of an animal with a body mass of 100 kg) in cantilever bending. The absolute value of the load applied is unimportant and was held constant across the models.

Subsequent beam models were generated of the same length (140 mm) and material properties. Ventral grooves of increasing depth were incorporated into the models (figure 3; electronic supplementary material, table S3). The outer radius of the beam was simultaneously increased, such that total volume of the model (1.099 e^4^ mm^3^) remained constant. Groove and outer radii were calculated using a custom Matlab script (see the electronic supplementary material, figure S1). All beam models were likewise loaded in ventrally directed cantilever bending with 9.8 N of force. All meshes comprised 4-node linear tetrahedral elements, and ranged in size from 1.1 to 1.7 million elements.

## 3. Results

**Figure 2.**
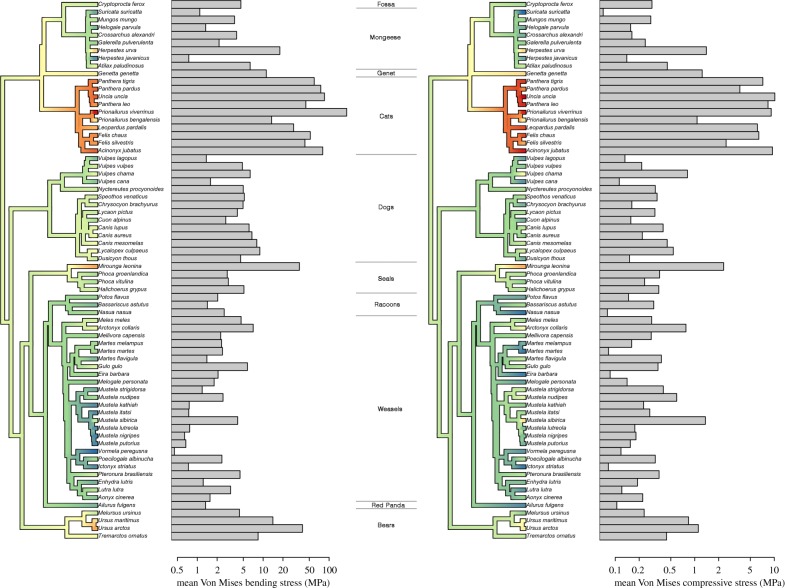
The phylogenetic distribution of mean Von Mises stress encountered during loading. Hot colours, high values of Von Mises stress; cool colours, low values of Von Mises stress. Consensus phylogeny sourced from the 10kTrees project (https://10ktrees.nunn-lab.org/Carnivora). (*a*) Mean Von Mises stress (MPa) under a bending load scenario. (*b*) Mean Von Mises stress (MPa) under a compressive loading scenario.

**Figure 3.**
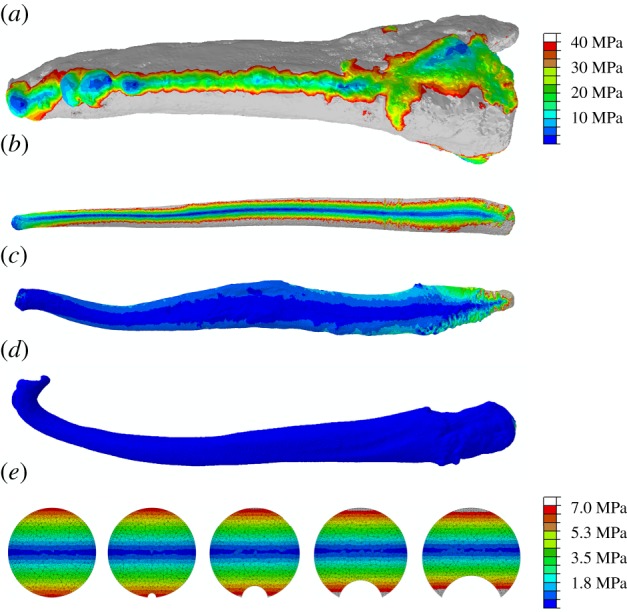
Von Mises stress maps. (*a–d*) Carnivoran bacula loaded under ventrally directed bending applied at the distal tip. Not to scale. (*a*) *Panthera tigris*; (*b*) *Ursus arctos*; (*c*) *Canis lupus*; (*d*) *Mustela putorius*. (*a–d*) Plotted on same colour scale, cool colours indicate *Mustela putorius* is considerably less stressed than *Panthera tigris* and *Ursus arctos*. Grey regions indicate stress has exceeded maximum value in legend. (*e*) Midshaft cross-sections through simple beam models of increasing groove depth. From left to right, groove depths of 0, 0.5, 1.5, 2.5 and 3.5 mm illustrated.

There is a strong phylogenetic signal in the distribution of mean *σ*_VM_ when bacula are loaded under bending (*λ* = 0.45, log*L* = −318.1, *p* < 0.001; figure 2*a*) and compression (*λ* = 0.63, log*L* = −133.0, *p* < 0.001; figure 2*b*). Bacula are consistently less stressed when loaded under compression (mean *σ*_VM_ 1.24 MPa) than cantilever bending (mean *σ*_VM_ 13.8 MPa). As expected given their diminutive size relative to body mass, felid bacula are weaker across the board (figures 2 and 3*a*). More surprisingly, ursid (figure 3*b*) and some pinniped bacula are also comparatively weak when loaded with an equivalent proportion of body mass (figure 2).

Under dorsoventral bending, the bacula of the Mustelinae (subfamily of mustelids characterized by extreme dorsal curvature of the baculum tip) are most resistant to deformation (figures 2*a* and 3*d*). By contrast, mustelids displaying less tip curvature (Melinae and Lutrinae) perform less well under a bending scenario. Canids perform similarly to mustelids when loaded under compression, yet are more susceptible to deformation under dorsoventral bending (figure 3*c*).

Type I error of the PGLS procedure is estimated at an acceptable 0.055 for the original dataset (*n* = 68), yet increases to a moderate 0.072 for the downsampled dataset (*n* = 47) for which intromission data also exist. The rate of detecting false positives, therefore, ranges from 5.5 to 7.2% depending upon the dataset and corresponding phylogeny. The power of the PGLS procedure to detect correlations of *β*_1_ = 0.75 and 0.90 is estimated to equal 1.00, and the likelihood of detecting false negatives is therefore extremely low.

**Figure 4.**
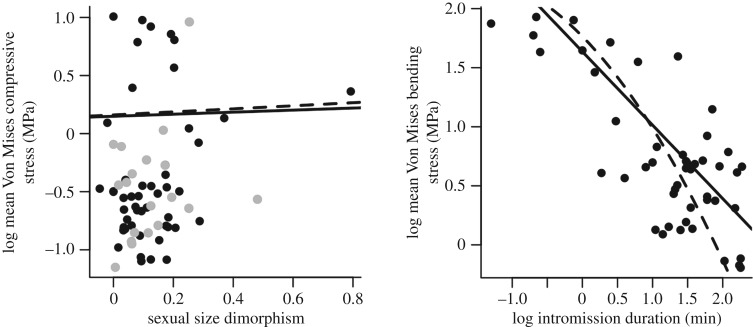
Phylogenetic regressions of mean *σ*_VM_ against life-history variables. (*a*) SSD was calculated as log(male body mass/female body mass). A weak positive correlation exists between SSD and mean *σ*_VM_ calculated under compression (*p* = 0.02, solid line) for the *n* = 68 original dataset (black plus grey circles). When downsampled to only include taxa for which intromission data also exists (black circles only, *n* = 47), this correlation becomes more significant (*p* = 0.005, dashed line). An outlier characterized by extremely high levels of SSD is identified as *Mirounga leonina*. When removed, both relationships become insignificant (*p* > 0.05). (*b*) A significant relationship does exist between intromission duration and mean bending *σ*_VM_ when modelled as a linear (*p* = 0.003, solid line) or quadratic (*p* = 0.0027, dashed line) regression.

A strong phylogenetic signal characterizes carnivoran intromission duration (*λ* = 0.79, log*L* = −39.2, *p* < 0.001; electronic supplementary material, figure S2), yet this signal is not significant within the SSD data (*λ* = 0.37, log*L* = 46.3, *p* = 0.10; electronic supplementary material, figure S2). A weak positive correlation is found between mean *σ*_VM_ under compression and SSD (compressive stress = 0.09 × SSD + 0.15, *t* = 2.48, *p* = 0.02, *n* = 68, AIC = −90.6; figure 4*a* solid line). In the light of the elevated type I error rate associated with PGLS on this dataset however, this result must be considered marginal. When rerun with a reduced sample size of *n* = 47 to match the dataset available for intromission duration, the relationship becomes more significant (compressive stress = 0.13 × SSD + 0.16, *t* = 2.97, *p* = 0.005, *n* = 47 dashed line). For this dataset, taxa that are characterized by high levels of SSD possess higher values of mean *σ*_VM_, i.e. have less ‘robust’ bacula under compressive loading. However, both relationships are highly sensitive to an outlier (*Mirounga leonina*, southern elephant seal) characterized by extreme values of SSD (figure 4*a*) and become insignificant when this taxon is removed (*n* = 67 dataset, *p* = 0.08; *n* = 46 dataset, *p* = 0.17). No significant relationship was identified between maximum baculum length and SSD (*p* = 0.55).

Mean *σ*_VM_ under bending is significantly correlated to intromission duration (bending stress = −0.62*intromission duration + 1.64, *t* = −3.12, *p* = 0.003, *n* = 47, AIC = 79.7; figure 4*b* solid line). Yet a quadratic regression model fits the data better according to AIC values (bending stress = −0.17*intromission duration^2^ ± 0.60*intromission duration + 1.76, *t* = −3.18, *p* = 0.0027, *n* = 47, AIC = 77.3; figure 4*b* dashed line). Taxa engaging in long bouts of copulation are characterized by low values of mean *σ*_VM_, i.e. possess more ‘robust’ bacula under dorsoventral bending. No significant relationship was identified between maximum baculum length and intromission duration (*p* = 0.26).

The results of our FEA beam simulations align with our understanding of beam theory. With increasing depth of groove, stress values at the base of the groove decrease (figure 3*e*). At a maximum groove depth of 22% of total diameter, stress values decrease by 35% at midshaft compared to a simple beam lacking a groove (electronic supplementary material, table S3). By contrast, *σ*_VM_ recorded on the dorsal surface on the beam increase by 12% in the presence of a well-developed groove (electronic supplementary material, table S3). Stress values also increase in the ventral ridges adjacent to the groove (figure 3).

## 4. Discussion

Bacula are found to be considerably less stressed when loaded in axial compression than dorsoventral bending (approximately by an order of magnitude, electronic supplementary material, table S1). This is unsurprising given the majority of carnivoran bacula approximate towards a ‘beam-like’ morphology. A notable exception is the Mustelinae, in which extreme distal curvature of the baculum results in compressive loads being applied off-axis, inducing considerable bending stresses (figure 2*b*). The poor performance of felid bacula under both scenarios is expected given their extremely diminutive size relative to mass. Indeed, the felid baculum is considered vestigial by some authors [36] and may not undergo significant loading during copulation. However, other bacula that outwardly appear large and robust (ursids and pinnipeds) also experience relatively high stresses under equivalent loading, complementing existing literature on fractured and healed bear and pinniped bacula [20,37]. This highlights the use of FEA when estimating biomechanical performance across a broad comparative sample, as predictions incorporate shape parameters such as aspect ratio and curvature, in addition to length.

A phylogenetic signal is detected in mean *σ*_VM_ calculated under bending (*λ* = 0.45) and compressive (*λ* = 0.63) loading. Given the role of sexual selection in shaping the baculum and the rapidity which with genitalic traits evolve, one might expect lower values of lambda to characterize bacula traits. As discussed elsewhere however [38], genital traits appear to evolve *rapidly enough* to be particularly useful in distinguishing closely related taxa compared to non-genital traits, yet still *slowly enough* to retain a phylogenetic signal reflecting higher-level groupings. Furthermore, the values of *σ*_VM_ here necessarily reflect the interaction between genital traits (gross baculum size incorporated into the FEA model) *and* non-genital traits (average male body mass with which bacula are loaded). It is therefore unsurprising that the resultant *σ*_VM_ values do contain a higher level of phylogenetic signal typically expected for genital traits alone.

The curved bacula of Mustelinae perform particularly well under ventrally directed bending loads (figure 2*a*). Owing to the dorsally directed distal curvature, applied ‘bending’ loads align closer to the neutral axis of the baculum, inducing comparatively more ‘compressive’ loads than occur in structures lacking curvature. By contrast, canid bacula experience relatively higher stresses than those of mustelids under dorsoventral bending. In addition to the lack of dorsoventral curvature, this might also be attributed to the deep urethral groove in caniform bacula. The groove results in comparatively less bony material distributed ventrally, hence lower values for second moment of area are likely in the direction of bending within this group.

SSD was found to correlate positively with *σ*_VM_ under compressive loading (figure 4*a*), albeit with marginal significance. This is contra to the relationship predicted by the vaginal friction hypothesis, in which taxa with high SSD are expected to possess more robust bacula under compression. When repeated on the subsampled data for which intromission data also exist, this relationship becomes more significant. The analysis presented here, therefore, suggests the carnivoran baculum does not represent an adaptation towards overcoming the initial problem of vaginal friction associated with SSD. If the southern elephant seal is considered an outlier in terms of extreme SSD, the relationship between SSD and *σ*_VM_ disappears entirely.

The degree to which average values of SSD are an adequate proxy for vaginal friction remains unclear. Comparatively little is known about female genital anatomy [39,40], and the potential role of lubrication of vaginal tissues is never accounted for. The dimensions of male soft tissue genital structures are only sporadically reported in carnivorans, presumably owing to inherent changes between flaccid and erect states. It is therefore quite feasible that genital soft tissue dimensions may scale to body mass differently between the sexes, and a simple metric of SSD fails to represent friction during intromission. Continuing and future efforts to document the three-dimensional soft tissue anatomy of vertebrates will go some way towards redressing this lack of data.

Mean *σ*_VM_ under dorsoventral bending was found to be significantly correlated to intromission duration (figure 4*b*). Carnivoran taxa engaging in prolonged copulation possess bacula that are more robust to bending. Interestingly, a curvilinear model better fits the relationship between bending *σ*_VM_ and intromission duration (figure 4*b*) compared to a linear model. This ‘tailing-off’ of intromission times may suggest factors beyond rigid mechanical support provide an upper limit to the length of copulation, such as soft tissue fatigue or the total energetic cost. Alternatively, the fact that bacula appear more robust to dorsoventral bending at longer intromission periods *than expected under a linear model* might be explained by the dominance of mustelids at longer copulation times. Mustelidae engage in extremely prolonged intromissions and possess exaggerated dorsally directed curvature of the baculum, resulting in high bending resistance in the dorsoventral direction.

Unsurprisingly, the relationship between *σ*_VM_ and intromission duration is characterized by a high level of noise. Values for copulation duration are sparsely reported, and are often based on anecdotal observations. Moreover, the period of time during mounting in which intromission is occurring can be extremely difficult to assess, and the number of thrusts per unit intromission time is likewise important yet non-trivial to measure. Some progress has been made towards quantifying the motor components of mammal copulation however. This includes the use of accelerometers to record thrusting activity, subcutaneous genital electrodes to quantify the duration of intromission and pressure transducers implanted in the seminal vesicles or corpus cavernosum to detect ejaculation [41]. Such studies understandably remain limited to laboratory species, however a future emphasis on non-invasively quantifying the kinematics of this basic organismal function across a broad suite of taxa would prove extremely informative.

In contrast to the significant relationships identified between FEA-derived metrics of baculum robustness versus SSD and intromission duration above, no significant relationships were found when solely considering length as a proxy for baculum performance. This is similar to previous findings when modelling SSD and intromission duration as continuous variables [11], suggesting the biomechanical performance of the baculum may not be reasonably estimated on the basis of length alone. This is unsurprising given the diverse array of forms taken by the carnivore baculum, and highlights the importance of taking a three-dimensional whole-organ approach to quantifying genital function. That baculum length does not correlate to intromission duration in carnivorans is counter to recent findings in which taxa were binned into ‘short’ and ‘long’ copulators however [5]. Given that both studies share an approximately equal sample size, this difference potentially stems from the methodological approach of treating intromission as a continuous versus discrete variable.

Our FEA models of speculative baculum morphs illustrate that stresses deep in the urethral groove are considerably reduced compared to those on the ventral margin (figure 3*e*). By lying within a deep groove, the urethra is placed closer to the neutral axis of bending. This supports the notion that the ventral groove acts not only to protect the urethra from irregular forceful impacts, but would also limit deformation occurring during typical copulation-induced bending of the glans. Proximal to the baculum, this may be accompanied by collagenous trabeculae within the corpus cavernosum acting to limit the expansion of the major hydrostatic system and protect the corpus spongiosum and urethra from further occlusion [42].

Furthermore, these results highlight the importance of incorporating *both* model average *σ*_VM_ metrics *and* the spatial distribution of *σ*_VM_ into our interpretation of biomechanical performance. Canid bacula on average display higher values of *σ*_VM_ under bending than mustelids, for example, yet the occurrence of a deep ventral groove suggests the canid urethra may experience relatively less deformation. Further experimental validation on cadaveric material, such as those initially conducted on bats [17], is necessary to confirm these findings.

We emphasize that the scenarios modelled using FEA are based entirely upon hypothetical loading regimes, and the extent to which the magnitude and direction of forces applied represent ‘realistic’ loads for bacula is unknown. As far as the authors are aware, the forces experienced by genitals during copulation have never been recorded, with the exception of intracavernous pressures within the cavernous bodies during erection [41]. Rather, we choose to load the bacula under two idealized scenarios frequently applied to simple beam models; axial compression and cantilever bending. Therefore, it must be recognized that our results are specific to those loading conditions. Mustelid bacula perform particularly well under dorsoventral bending owing to the occurrence of dorsally directed tip curvature, for example, yet the *in copula* forces experienced probably combine some unknown degree of compression, bending and torsion. Likewise, the baculum is modelled as experiencing axial compression during initial intromission. Yet this overlooks the complex interactions of soft tissues possibly experiencing shear [21]. This will particularly be the case in taxa characterized by the presence of keratinous spines (for example, felids and the fossa) on the surface of the glans, although the extent of shear will vary with the direction of spines. Furthermore, any potential movement of the penis relative to the pelvis via intrinsic musculature remains unquantified. Future *in vivo* studies of vertebrate copulation using imaging modalities such as magnetic resonance imaging [43] and X-ray reconstruction of moving motion may future illuminate the loading regimes to which genitals are subjected.

The magnitude of loads imparted on the baculum during copulation is unknown, yet the occurrence of healed fractures in osteological collections [20] suggests they can be loaded to failure. Here we choose to apply only 1% of male body mass, resulting in average stresses of 1.24 MPa and 13.8 MPa for compression and bending, respectively. Assuming a yield stress similar to that of cortical bone in the human tibia (180 MPa under compression, 128 MPa under tension [44]) results in safety factors of the order of magnitude 10–150. In reality, yield stress decreases alongside Young's modulus [45], and the low recorded values for Young's modulus in the canid baculum [25] imply yield stress and safety factors may be considerably lower than those of human cortical bone. Hence, when loaded with only 1% of body mass (particularly under bending), baculum safety factors already appear to be approaching those known to characterize vertebrate limb bones during locomotion [46].

This assumes a constant value for Young's modulus across the sample, yet mechanical-testing data are lacking. Given the range of Young's modulus values within carnivoran limb cortical bone (14–22 GPa; [47]), variability in baculum material properties also appears likely. The phylogenetic distribution of material properties, and their potential for negating/reinforcing the trends identified herein, therefore remains unknown. Furthermore, the assumption of a constant proportion of body mass being applied to the baculum is probably a simplification. Carnivorans vary considerably in their mating behaviour; female cats lie down and remain relatively still during copulation, mustelids often engage in vigorous rolling, while dogs reposition themselves tail-to-tail during the copulatory tie. It, therefore, seems likely that the magnitude and direction of forces experienced during copulation differ markedly across Carnivora.

## 5. Conclusion

For the first time, to our knowledge, we take a three-dimensional computational simulation approach to testing biomechanical hypotheses for the role of the mammalian baculum. Carnivoran taxa engaging in prolonged copulation are found to possess bacula that are significantly more resistant to dorsoventral bending. Furthermore, the addition of a ventrally located groove considerably reduces stress within the region of the urethra. Our results support the notion that the size and shape of the carnivoran baculum have evolved in response to selective pressures on the duration of copulation and the protection of the urethra. Future research will focus on quantifying associated soft-tissue geometries in both males and females in order to take a whole ‘organ system’ approach to reproductive mechanics, and on developing methodologies to successfully capture the kinematics of mating.

## Supplementary Material

Specimen information and groove calculations

## Supplementary Material

MATLAB code for groove calculations

## Data accessibility

The datasets supporting this article have been uploaded as part of the electronic supplementary material.
