# Supplementary material for: Testing hypotheses for the function of the carnivoran baculum using finite-element analysis
Source: Proc Biol Sci. 2018 Sep 19;285(1887):20181473. doi: 10.1098/rspb.2018.1473 (PMC6170803; doi:10.1098/rspb.2018.1473)
Supplement: Specimen information and groove calculations [file rspb20181473supp1.docx]

**Supplementary Material**

**Table S1. Specimen list and associated life history data.** NMS, National Museum of Scotland; NHM, Natural History Museum, London; UoM, University of Manchester Life Sciences Teaching Collection.

| Taxa | Museum | Male mass (kg) | Average male mass (kg) | Average female mass (kg) | Intromission (mins) |
| --- | --- | --- | --- | --- | --- |
| *Acinonyx_jubatus* | NMS |  | 43.7^1^ | 35^1^ | 0.75^2^ |
| *Ailurus_fulgens* | NMS |  | 5.4^3^ | 5.2^3^ | 25^4^ |
| *Aonyx_cinerea* | NMS |  | 3.47^5^ | 3.20^5^ | 30^6^ |
| *Arctonyx_collaris* | NHM |  | 8.93^7^ | 8.40^7^ |  |
| *Atilax_paludinosus* | NHM |  | 2.96^8^ | 2.56^8^ |  |
| *Bassariscus_astutus* | NHM |  | 1.32^3^ | 0.94^3^ | 17^9^ |
| *Canis_aureus* | NHM |  | 12^10^ | 10^10^ | 11.5^11^ |
| *Canis_lupus* | UoM |  | 45^10^ | 41^10^ | 120^12^ |
| *Canis_mesomelas* | NHM |  | 8.2^10^ | 7.1^10^ |  |
| *Chrysocyon_brachyurus* | NMS |  | 23^10^ | 20^10^ | 31^13^ |
| *Crossarchus_alexandri* | NHM |  | 1.55^3^ | 1.1^14^ |  |
| *Cryptoprocta_ferox* | NMS | 10.35* | 7.40^15^ | 6.10^15^ | 188^16^ |
| *Cuon_alpinus* | NMS | 6.2* | 17.5^10^ | 11.5^10^ | 20^17^ |
| *Dusicyon_thous* | NHM |  | 7.0^10^ | 6.4^10^ | 8^18^ |
| *Eira_barbara* | NHM |  | 4.32^3^ | 3.50^3^ | 35^19^ |
| *Enhydra_lutris* | NMS |  | 32.8^20^ | 21.5^20^ | 14^21^ |
| *Felis_chaus* | NMS | 5.3* | 8.07^3^ | 5.05^3^ | 2.5^22^ |
| *Felis_silvestris* | NMS |  | 5.01^1^ | 4.33^1^ | 0.25^23^ |
| *Galerella_pulverulenta* | NHM |  | 0.91^3^ | 0.68^3^ |  |
| *Genetta_genetta* | NHM |  | 1.87^3^ | 1.95^3^ | 3^24^ |
| *Gulo_gulo* | NHM |  | 14.2^20^ | 9.41^20^ | 27^12^ |
| *Halichoerus_grypus* | NMS |  | 250.4^25^ | 187.21^25^ | 30^26^ |
| *Helogale_parvula* | NHM |  | 0.25^3^ | 0.23^3^ | 11^27^ |
| *Herpestes_javanicus* | NHM |  | 0.69^1^ | 0.53^1^ | 0.83^28^ |
| *Herpestes_semitorquatus* | NHM |  | 2.2^29,A^ |  |  |
| *Herpestes_urva* | NHM |  | 2.6^3,A^ |  |  |
| *Ictonyx_striatus* | NHM |  | 0.96^30^ | 0.64^30^ | 106^31^ |
| *Leopardus_pardalis* | NMS | 10.8* | 13^3^ | 10.8^3^ | 1.5^22^ |
| *Lutra_lutra* | NMS |  | 9.60^3^ | 6.75^3^ | 22.5^21^ |
| *Lycalopex_culpaeus* | NHM |  | 11.7^32^ | 7.82^32^ |  |
| *Lycalopex_griseus* | NHM |  | 4.6^10^ | 4.2^10^ |  |
| *Lycaon_pictus* | NMS |  | 25.0^10^ | 25.0^10^ | 1.87^33^ |
| *Lyncodon_patagonicus* | NHM |  | 0.23^3,A^ |  |  |
| *Martes_flavigula* | NHM |  | 2.88^34^ | 2.60^34^ |  |
| *Martes_martes* | NMS | 1.83* | 1.36^35^ | 1.02^35^ | 60^21^ |
| *Martes_melampus* | NMS | 1.55* | 1.50^36^ | 1.00^36^ |  |
| *Meles_meles* | NMS |  | 11.6^20^ | 10.1^20^ | 90^37^ |
| *Mellivora_capensis* | NHM |  | 9.70^1^ | 6.20^1^ |  |
| *Melogale_everetti* | NHM |  | 2.00^38^ |  |  |
| *Melogale_moschata* | NHM |  | 1.42^1^ | 1.15^1^ |  |
| *Melogale_orientalis* | NHM |  | 2.00^38^ |  |  |
| *Melogale_personata* | NHM |  | 2.00^39^ | 1.70^39^ |  |
| *Melursus_ursinus* | NMS |  | 75^40^ | 58^40^ | 35^41^ |
| *Mirounga_leonina* | NMS |  | 3510^25^ | 565.7^25^ | 6.2^42,B^ |
| *Mungos_mungo* | NHM |  | 1.46^43^ | 1.35^43^ | 4^44^ |
| *Mustela_itatsi* | NHM |  | 0.54^45^ | 0.18^45^ |  |
| *Mustela_kathiah* | NHM |  | 0.36^3^ | 0.20^3^ |  |
| *Mustela_lutreola* | NMS |  | 0.85^1^ | 0.44^1^ | 180^46^ |
| *Mustela_nigripes* | NMS |  | 1.50^1^ | 1.35^1^ | 180^21^ |
| *Mustela_nudipes* | NHM |  | 0.65^3,A^ |  |  |
| *Mustela_putorius* | NMS |  | 1.11^3^ | 0.69^3^ | 172^47^ |
| *Mustela_sibirica* | NHM |  | 0.80^45^ | 0.35 | 160^48^ |
| *Mustela_strigidorsa* | NHM |  | 0.60^49,A^ |  |  |
| *Nasua_nasua* | NMS |  | 4.28^3^ | 3.45^3^ | 60^50,C^ |
| *Nyctereutes_procyonoides* | NMS |  | 7.5^10^ | 7.5^10^ | 10^21^ |
| *Panthera_leo* | NMS | 140* | 185.9 | 139.5^3^ | 1^51^ |
| *Panthera_pardus* | NMS |  | 48.1^3^ | 30.2^3^ | 0.05^52^ |
| *Panthera_tigris_sumatrae* | NMS |  | 131.2^53^ | 84.3^53^ | 0.2^52^ |
| *Phoca_groenlandica* | NMS |  | 102.6^25^ | 98.6^25^ |  |
| *Phoca_vitulina* | NMS |  | 75.0^25^ | 63.3^25^ | 21^54^ |
| *Poecilogale_albinucha* | NHM |  | 0.33^3^ | 0.20^3^ | 78^31^ |
| *Potos_flavus* | NMS |  | 3.53^55^ | 3.27^55^ | 150^55^ |
| *Prionailurus_bengalensis* | NMS | 5* | 3.3^3^ | 2.25^3^ |  |
| *Prionailurus_viverrinus* | NMS | 9.9* | 11.3^1^ | 6.3^1^ |  |
| *Pteronura_brasiliensis* | NMS |  | 30.0^20^ | 24.0^20^ | 30^56^ |
| *Speothos_venaticus* | NMS | 8.97* | 9.0^10^ | 10.0^10^ | 52^57^ |
| *Suricata_suricatta* | NHM |  | 0.73^1^ | 0.72^1^ |  |
| *Tremarctos_ornatus* | NMS | 106* | 106* | 71^58,D^ | 60^59^ |
| *Uncia_uncia* | NMS | 42* | 32.5^1^ | 32.5^1^ | 0.22^52^ |
| *Ursus_arctos* | NHM |  | 200^60^ | 111.9^3^ | 23^12^ |
| *Ursus_maritimus* | NMS |  | 360^21^ | 187^3^ | 71^61^ |
| *Vormela_peregusna* | NMS |  | 0.43^62^ | 0.37^62^ |  |
| *Vulpes_cana* | NMS | 1.6* | 0.96^3^ | 0.83^3^ |  |
| *Vulpes_chama* | NHM |  | 2.60^10^ | 2.60^10^ |  |
| *Vulpes_lagopus* | NMS |  | 3.80^10^ | 3.10^10^ | 37^63^ |
| *Vulpes_vulpes* | NMS |  | 6.70^10^ | 5.40^10^ | 40^12^ |

1. Iossa, G., Soulsbury, C. D., Baker, P. J. & Harris, S. Sperm competition and the evolution of testes size in terrestrial mammalian carnivores. *Funct. Ecol.* **22,** 655–662 (2008).

2. Frank, J. & Saffoe, C. *Breeding Management Strategy for Cheetahs (Acinonyx jubatus) at the Smithsonian’s National Zoological Park*. (2005).

3. Silva, M. & Downing, J. A. *CRC Handbook of Mammalian Body Masses*. (CRC Press, 1995).

4. Roberts, M. S. & Kessler, D. S. Reproduction in Red pandas, Ailurus fulgens (Carnivora: Ailuropodidae). *J. Zool.* **188,** 235–249 (1979).

5. Lemasson, A., Mikus, M.-A., Blois-Heulin, C. & Lodé, T. Vocal repertoire, individual acoustic distinctiveness, and social networks in a group of captive Asian small-clawed otters (Aonyx cinerea). *J. Mammal.* **95,** 128–139 (2014).

6. Heap, C. J., Wright, L. & Andrews, L. *Summary of Husbandry Guidelines for Asian Small-clawed Otters in Captivity.* *IUCN/SSC OTTER Specialist Group, Otters in Captivity Task Force* (2008).

7. Parker, C. Birth, care and development of Chinese hog badgers at Metro Toronto Zoo. *Int. Zoo Yearb.* **19,** 182–185 (1977).

8. Baker, C. M. Atilax paludinosus. *Mamm. Species* **408,** 1–6 (1992).

9. Poglayen-Neuwall, I. Management and breeding of the Ringtail or Cacomistle Bassariscus astutus in captivity. *Int. Zoo Yearb.* **26,** 276–280 (1987).

10. Palmqvist, P., Mendoza, M., Arribas, A. & Gröcke, D. R. Estimating the body mass of Pleistocene canids: discussion of some methodological problems and a new ‘taxon free’ approach. *Lethaia* **35,** 358–360 (2002).

11. Golani, I. & Mendelssohn, H. Sequences of Precopulatory Behavior of the Jackal (Canis aureus). *Behaviour* **38,** 169–192 (1971).

12. Larivière, S. & Ferguson, S. H. On the evolution of the mammalian baculum: Vaginal friction, prolonged intromission or induced ovulation? *Mamm. Rev.* **32,** 283–294 (2002).

13. Weinhardt, D. & Rodden, M. in *Husbandry manual for the Maned Wolf (Chrysocyon brachyurus)* (eds. Fletchall, N. B., Rodden, M. & Taylor, S.) 29–41 (SSP Husbandry Manual, 1995). doi:10.1016/B978-1-4377-1986-4.00058-5

14. Goldman, C. A. Systematic revision of the African mongoose genus Crossarchus (Mammalia: Viverridae). *Can. J. Zool.* **62,** 1618–1630 (1984).

15. Hawkins, C. E. Behaviour and ecology of the fossa, Cryptoprocta ferox (Carnivora: Viverridae) in a dry deciduous forest, western Madagascar. (University of Aberdeen, 1998).

16. Hawkins, C. E. & Racey, P. A. A novel mating system in a solitary carnivore: The fossa. *J. Zool.* **277,** 196–204 (2009).

17. Sosnovskii, I. P. Breeding the Red dog or dhole Cuon alpinus at Moscow Zoo. *Int. Zoo Yearb.* **7,** 120–122 (1967).

18. Brady, C. A. Reproduction, growth and parental care in crag- eating foxes (Cerdocyon thous) at the National Zoological Park, Washington. *Int. Zoo Yearb.* **18,** 130–134 (1978).

19. Poglayen-Neuwall, I. Copulatory behavior, gestation and parturition of the tayra (Eira barbara L., 1758). *Zeitschrift fur Saugetierkd.* **40,** 176–189 (1975).

20. Weckerly, F. W. Sexual-size dimorphism: Influence of Mass and Mating Systems in the Most Dimorphic Mammals. *J. Mammal.* **79,** 33–52 (1998).

21. Dixson, A., Nyholt, J. & Anderson, M. A positive relationship between baculum length and prolonged intromission patterns in mammals. *Acta Zool. Sin.* **50,** 490–503 (2004).

22. Mellen, J. D. A Comparative Analysis of Scent-Marking, Social and Reproductive Behavior in 20 Species of Small Cats (Felis). *Am. Zool.* **33,** 151–166 (1993).

23. Dixson, A. F. Baculum length and copulatory behaviour in carnivores and pinnipeds (Grand Order Ferae). *J. Zool. Soc. London* **235,** 67–76 (1995).

24. Lariviere, S. & Calzada, J. Genetta genetta. *Mamm. Species* **680,** 1–6 (2001).

25. Fitzpatrick, J. L., Almbro, M., Gonzalez-Voyer, A., Kolm, N. & Simmons, L. W. Male contest competition and the coevolution of weaponry and testes in pinnipeds. *Evolution (N. Y).* **66,** 3595–3604 (2012).

26. Dixson, A. F. & Anderson, M. J. Sexual behavior, reproductive physiology and sperm competition in male mammals. *Physiol. Behav.* **83,** 361–371 (2004).

27. Rood, J. P. Mating relationships and breeding suppression in the dwarf mongoose. *Anim. Behav.* **28,** 143–150 (1980).

28. Datta, A. K. Notes on Mating Behaviour of Small Asian Mongoose Herpestes javanicus at Jahangirnagar University Campus, Dhake. *Zoo’s Print* **XXIX,** 7–8 (2015).

29. Okie, J. G. & Brown, J. H. Niches, body sizes, and the disassembly of mammal communities on the Sunda Shelf islands. *Proc. Natl. Acad. Sci.* **106,** 19679–19684 (2009).

30. Lariviere, S. Ictonyx striatus. *Mamm. Species* **698,** 1–5 (2002).

31. Rowe-Rowe, D. T. Reproduction and post-natal development of South African mustelines (Carnivora: Mustelidae). *Zool. Africana* **13,** 103–114 (1978).

32. Johnson, W. E. & Franklin, W. L. Spatial resource partitioning by sympatric grey fox (Dusicyon griseus) and culpeo fox (Dusicyon culpaeus) in southern Chile. *Can. J. Zool.* **72,** 1788–1793 (1994).

33. Van Heerden, J. The role of integumental glands in the social and mating behaviour of the hunting dog Lycaon pictus (Temminck, 1820). *Onderstepoort J. Vet. Res.* **48,** 19–21 (1981).

34. Grassman, L. I., Janecka, J. E., Austin, S. C., Tewes, M. E. & Silvy, N. J. Chemical immobilization of free-ranging dhole (Cuon alpinus), binturong (Arctictis binturong), and yellow-throated marten (Martes flavigula) in Thailand. *Eur. J. Wildl. Res.* **52,** 297–300 (2006).

35. Ferguson, S. H. & Lariviere, S. Are Long Penis Bones an Adaption to High Latitude Snowy Environments? *Oikos* **105,** 255–267 (2004).

36. Tatara, M. Notes on the Breeding Ecology and Behavior of Japanese Martens on Tsushima Islands, Japan. *J. Mamm. Soc. Japan* **19,** 67–74 (1994).

37. Yamaguchi, N., Dugdale, H. L. & MacDonald, D. W. Female receptivity, embryonic diapause and superfoetation in the European badger (Meles meles): implications for the reproductive tactics of males and females. *Q. Rev. Biol.* **81,** 33–48 (2006).

38. Johnson, D. D. P., Macdonald, D. W. & Dickman, A. M. Y. J. An analysis and review of models of the sociobiology of the Mustelidae. *Mamm. Rev.* **30,** 171–196 (2000).

39. Islam, M. A., Chowdhury, G. W. & Belant, J. L. First record of the Large-toothed Ferret Badger in Bangladesh. *Small Carniv. Conserv.* **39,** 41–42 (2008).

40. Ratnayeke, S., van Manen, F. T. & Padmalal, U. K. G. K. Home Ranges and Habitat Use of Sloth Bears Melursus Ursinus Inornatus in Wasgomuwa National Park, Sri Lanka. *Wildlife Biol.* **13,** 272–284 (2007).

41. Joshi, A. R., Smith, J. L. D. & Garshelis, D. L. Sociobiology of the myrmecophagous sloth bear in Nepal. *Can. J. Zool.* **77,** 1690–1704 (1999).

42. Leboeuf, B. J. Sexual Behavior in the Northern Elephant Seal Mirounga Angustirostris. *Behaviour* **41,** 1–26 (1972).

43. Otali, E. & Gilchrist, J. S. The effcts of refuse feeding on body condition, reproduction, and survial of banded mongooses. *J. Mammal.* **85,** 491–497 (2004).

44. Cant, M. A., Otali, E. & Mwanguhya, F. Fighting and mating between groups in a cooperatively breeding mammal, the banded mongoose. *Ethology* **108,** 541–555 (2002).

45. Sasaki, H. *et al.* Factors Affecting the Distribution of the Japanese Weasel Mustela itatsi and the Siberian Weasel M . sibirica in Japan. *Mammal Study* **39,** 133–139 (2014).

46. Youngman, P. M. Mustela lutreola. *Mamm. Species* **362,** 1–3 (1990).

47. Miller, B. J. & Anderson, S. H. Failure of fertilization following abbreviated copulation in the ferret (Mustela putorius furo). *J. Exp. Zool.* **249,** 85–89 (1989).

48. Amstislavsky, S. Reproductive Biology and Embryo Technology in Mustelidae. *Kuopio University Publications* (University of Kuopio, 2009).

49. Streicher, U., Duckworth, J. W. & Robichaud, W. G. Further Records of Stripe-backed Weasel Mustela strigidorsa from Lao PDR. *Trop. Nat. Hist.* **10,** 199–203 (2010).

50. Hass, C. C. & Roback, J. F. Copulatory behavior of White-Nosed Coatis. *Southwest. Assoc. Nat.* **45,** 329–331 (2000).

51. Dixson, A. F. Baculum length and copulatory behaviour in carnivores and pinnipeds (Grand Order Ferae). *J. Zool.* **235,** 67–76 (1995).

52. Lanier, D. L. & Dewsbury, D. A. A Quantitative Study of Copulatory Behaviour of Large Felidae. *Behav. Processes* **1,** 327–333 (1976).

53. Narayan, E. J. *et al.* Faecal cortisol metabolites in Bengal (Panthera tigris tigris) and Sumatran tigers (Panthera tigris sumatrae). *Gen. Comp. Endocrinol.* **194,** 318–325 (2013).

54. Allen, S. G. Mating Behavior in the Harbor Seal. *Mar. Mammal Sci.* **1,** 84–87 (1985).

55. Kays, R. W. & Gittleman, J. L. The social organization of the kinkajou Potos flavus (Procyonidae). *J. Zool.* **253,** 491–504 (2001).

56. Hagenbeck, C. & Wunnemann, K. Breeding the Giant otter Pteronura brasiliensis at Carl Hagenbecks Tierpark. *Int. Zoo Yearb.* **31,** 240–245 (1992).

57. DeMatteo, K. E., Porton, I. J., Kleiman, D. G. & Asa, C. S. The Effect of the Male Bush Dog (Speothos Venaticus) on the Female Reproductive Cycle. *J. Mammal.* **87,** 723–732 (2006).

58. García-Rangel, S. Andean bear Tremarctos ornatus natural history and conservation. *Mamm. Rev.* **42,** 85–119 (2012).

59. Bloxam, Q. Breeding the Spectacled bear Tremarctos ornatus at Jersey Zoo. *Int. Zoo Yearb.* **17,** 158–161 (1977).

60. Ramm, S. A. Sexual Selection and Genital Evolution in Mammals: A Phylogenetic Analysis of Baculum Length. *Am. Nat.* **169,** 360–369 (2007).

61. Ferguson, S. H. Polar Bears: The Natural History of a Threatened Species by Ian Stirling. *Arctic* **1,** 107–108 (2012).

62. Gorsuch, W. A. & Larivière, S. Vormela peregusna. *Mamm. Species* **779,** 1–5 (2005).

63. Valberg Nordrum, N. M. Effect of Inbreeding on Reproductive Performance in Blue Fox (Alopex lagopus) Vixens. *Acta Agric. Scand. A Anim. Sci.* **44,** 214–221 (1994).

*Associated body masses from zoo cadavers recorded at/near time of death.

^A^Sex specific data not available

^B^Data pertains to *M. angustirostris* matings in the water, as opposed to land

^C^Data relates to *N. narica*

^D^Female mass based upon study ascertion that females are approximately 2/3^rd^ the size of males

N.B. For body mass values sourced from the CRC Handbook of Mammalian Body Masses, values here represent sex-specific averages of those presented in the book.

**Table S2. CT scanning parameters and finite element results.**

| Taxa | Location | Resolution (mm) | kV | uA | Element number | Bending mean σ_VM_ | Compression mean σ_VM_ |
| --- | --- | --- | --- | --- | --- | --- | --- |
| *Acinonyx_jubatus* | MXIF | 0.0070 | 100 | 170 | 1938362 | 80.05 | 9.480 |
| *Ailurus_fulgens* | MXIF | 0.0324 | 75 | 80 | 3597129 | 1.336 | 0.105 |
| *Aonyx_cinerea* | MXIF | 0.0324 | 75 | 80 | 1676244 | 1.560 | 0.222 |
| *Arctonyx_collaris* | NHM | 0.0234 | 150 | 160 | 2640671 | 7.057 | 0.775 |
| *Atilax_paludinosus* | NHM | 0.0157 | 140 | 150 | 1223952 | 6.343 | 0.451 |
| *Bassariscus_astutus* | NHM | 0.0311 | 140 | 150 | 2623776 | 1.422 | 0.305 |
| *Canis_aureus* | NHM | 0.0480 | 150 | 160 | 2296524 | 6.754 | 0.219 |
| *Canis_lupus* | MXIF | 0.0400 | 75 | 80 | 2498853 | 6.112 | 0.400 |
| *Canis_mesomelas* | NHM | 0.0311 | 140 | 150 | 4597342 | 8.010 | 0.450 |
| *Chrysocyon_brachyurus* | MXIF | 0.0400 | 75 | 80 | 1855043 | 4.955 | 0.162 |
| *Crossarchus_alexandri* | NHM | 0.0157 | 140 | 150 | 4893855 | 3.955 | 0.162 |
| *Cryptoprocta_ferox* | MXIF | 0.0324 | 75 | 80 | 1437063 | 4.593 | 0.290 |
| *Cuon_alpinus* | MXIF | 0.0324 | 75 | 80 | 1928119 | 2.708 | 0.157 |
| *Dusicyon_thous* | NHM | 0.0480 | 140 | 150 | 2083094 | 4.549 | 0.151 |
| *Eira_barbara* | NHM | 0.0480 | 140 | 150 | 2490487 | 2.067 | 0.086 |
| *Enhydra_lutris* | MXIF | 0.0500 | 100 | 90 | 2140495 | 1.229 | 0.190 |
| *Felis_chaus* | MXIF | 0.0070 | 100 | 90 | 1772980 | 51.81 | 6.394 |
| *Felis_silvestris* | MXIF | 0.0070 | 100 | 170 | 474315 | 42.94 | 2.478 |
| *Galerella_pulverulenta* | NHM | 0.0157 | 140 | 150 | 4214784 | 2.142 | 0.238 |
| *Genetta_genetta* | NHM | 0.0157 | 140 | 150 | 2611880 | 11.15 | 1.239 |
| *Gulo_gulo* | NHM | 0.0480 | 150 | 160 | 2454775 | 5.782 | 0.345 |
| *Halichoerus_grypus* | MXIF | 0.0500 | 120 | 160 | 3598327 | 5.110 | 0.353 |
| *Helogale_parvula* | NHM | 0.0157 | 140 | 150 | 1219846 | 1.342 | 0.156 |
| *Herpestes_javanicus* | NHM | 0.0157 | 140 | 150 | 3889643 | 0.743 | 0.140 |
| *Herpestes_semitorquatus* | NHM | 0.0157 | 140 | 150 | 3286740 | 5.065 | 0.179 |
| *Herpestes_urva* | NHM | 0.0157 | 140 | 150 | 1645434 | 17.93 | 1.400 |
| *Ictonyx_striatus* | NHM | 0.0480 | 150 | 160 | 1813810 | 0.731 | 0.082 |
| *Leopardus_pardalis* | MXIF | 0.0070 | 100 | 170 | 831519 | 28.91 | 6.127 |
| *Lutra_lutra* | MXIF | 0.0400 | 75 | 80 | 3796976 | 3.199 | 0.121 |
| *Lycalopex_culpaeus* | NHM | 0.0480 | 150 | 160 | 1424724 | 8.911 | 0.537 |
| *Lycaon_pictus* | MXIF | 0.0400 | 75 | 80 | 3886283 | 4.062 | 0.316 |
| *Lyncodon_patagonicus* | NHM | 0.0233 | 150 | 160 | 1701256 | 1.109 | 0.048 |
| *Martes_flavigula* | NHM | 0.0480 | 150 | 160 | 2141252 | 1.399 | 0.380 |
| *Martes_martes* | MXIF | 0.0324 | 75 | 80 | 1352581 | 2.408 | 0.083 |
| *Martes_melampus* | MXIF | 0.0324 | 75 | 80 | 1010851 | 2.338 | 0.161 |
| *Meles_meles* | MXIF | 0.0324 | 75 | 80 | 1222700 | 4.623 | 0.287 |
| *Mellivora_capensis* | NHM | 0.0480 | 150 | 160 | 4148499 | 2.266 | 0.283 |
| *Melogale_everetti* | NHM | 0.0311 | 140 | 150 | 1064883 | 3.050 | 0.414 |
| *Melogale_orientalis* | NHM | 0.0480 | 140 | 150 | 1824250 | 1.870 | 0.082 |
| *Melogale_personata* | NHM | 0.0480 | 140 | 150 | 1255368 | 1.800 | 0.141 |
| *Melursus_ursinus* | MXIF | 0.0500 | 100 | 90 | 2959114 | 4.367 | 0.231 |
| *Mirounga_leonina* | MXIF | 0.0500 | 120 | 160 | 4317706 | 35.42 | 2.305 |
| *Mungos_mungo* | NHM | 0.0157 | 140 | 150 | 4035227 | 3.677 | 0.280 |
| *Mustela_itatsi* | NHM | 0.0311 | 140 | 150 | 1313569 | 0.741 | 0.272 |
| *Mustela_kathiah* | NHM | 0.0233 | 150 | 160 | 493725 | 0.754 | 0.227 |
| *Mustela_lutreola* | MXIF | 0.0324 | 75 | 80 | 1116972 | 0.767 | 0.176 |
| *Mustela_nigripes* | MXIF | 0.0324 | 75 | 80 | 1287366 | 0.640 | 0.182 |
| *Mustela_nudipes* | NHM | 0.0311 | 140 | 150 | 1232178 | 2.459 | 0.593 |
| *Mustela_putorius* | MXIF | 0.0400 | 75 | 80 | 1598726 | 0.670 | 0.155 |
| *Mustela_sibirica* | NHM | 0.0233 | 150 | 160 | 675361 | 4.106 | 1.360 |
| *Mustela_strigidorsa* | NHM | 0.0311 | 140 | 150 | 1018951 | 1.185 | 0.402 |
| *Nasua_nasua* | MXIF | 0.0324 | 75 | 80 | 4818202 | 2.560 | 0.080 |
| *Nyctereutes_procyonoides* | MXIF | 0.0324 | 75 | 80 | 1331078 | 4.986 | 0.319 |
| *Panthera_leo* | MXIF | 0.0070 | 100 | 170 | 967987 | 44.34 | 8.340 |
| *Panthera_pardus* | MXIF | 0.0070 | 100 | 170 | 1552446 | 74.76 | 3.690 |
| *Panthera_tigris_sumatrae* | MXIF | 0.0070 | 120 | 130 | 421036 | 59.42 | 7.182 |
| *Phoca_groenlandica* | MXIF | 0.0500 | 120 | 160 | 1305375 | 2.848 | 0.361 |
| *Phoca_vitulina* | MXIF | 0.0500 | 120 | 160 | 3014534 | 2.956 | 0.234 |
| *Poecilogale_albinucha* | NHM | 0.0233 | 150 | 160 | 718126 | 2.365 | 0.319 |
| *Potos_flavus* | MXIF | 0.0324 | 75 | 80 | 1528489 | 2.043 | 0.147 |
| *Prionailurus_bengalensis* | MXIF | 0.0070 | 100 | 170 | 4331231 | 13.45 | 1.069 |
| *Prionailurus_viverrinus* | MXIF | 0.0070 | 100 | 170 | 288326 | 185.0 | 9.133 |
| *Pteronura_brasiliensis* | MXIF | 0.0400 | 75 | 80 | 3209414 | 4.455 | 0.356 |
| *Speothos_venaticus* | MXIF | 0.0400 | 75 | 80 | 5527826 | 5.171 | 0.336 |
| *Suricata_suricatta* | NHM | 0.0157 | 140 | 150 | 593060 | 1.092 | 0.071 |
| *Tremarctos_ornatus* | MXIF | 0.0500 | 100 | 90 | 3266872 | 8.376 | 0.442 |
| *Uncia_uncia* | MXIF | 0.0070 | 100 | 170 | 669269 | 84.96 | 10.16 |
| *Ursus_arctos* | NHM | 0.0665 | 140 | 150 | 3911318 | 39.40 | 1.109 |
| *Ursus_maritimus* | MXIF | 0.0500 | 100 | 90 | 1458871 | 14.07 | 0.836 |
| *Vormela_peregusna* | MXIF | 0.0324 | 75 | 80 | 1088572 | 0.449 | 0.118 |
| *Vulpes_cana* | MXIF | 0.0324 | 75 | 80 | 2182004 | 1.583 | 0.112 |
| *Vulpes_chama* | NHM | 0.0233 | 150 | 160 | 1404688 | 6.348 | 0.808 |
| *Vulpes_lagopus* | MXIF | 0.0400 | 75 | 80 | 937493 | 1.369 | 0.132 |
| *Vulpes_vulpes* | MXIF | 0.0324 | 75 | 80 | 2365703 | 4.830 | 0.215 |

| **Baculum *r* (mm)** | **Groove *r* (mm)** | **% depth** | **Dorsal max. σ_VM_ (MPa)** | **Groove max. σ_VM_ (MPa)** |
| --- | --- | --- | --- | --- |
| 5 | 0 | 0 | 6.86 | 6.86 |
| 5.01 | 0.5 | 6 | 6.92 | 6.30 |
| 5.05 | 1.0 | 9 | 7.01 | 5.68 |
| 5.10 | 1.5 | 12 | 7.13 | 5.27 |
| 5.18 | 2.0 | 16 | 7.28 | 4.98 |
| 5.27 | 2.5 | 18 | 7.43 | 4.73 |
| 5.38 | 3.0 | 20 | 7.55 | 4.70 |
| 5.50 | 3.5 | 22 | 7.69 | 4.46 |

**Table S3. Finite element results of grooved beam analysis.** *r*, radius. Reported Von Mises stresses were extracted from midshaft at the dorsal surface and at the maximum depth of the ventral groove.

**Figure S1. Calculation of groove and outer radii dimensions of simplified beam model**


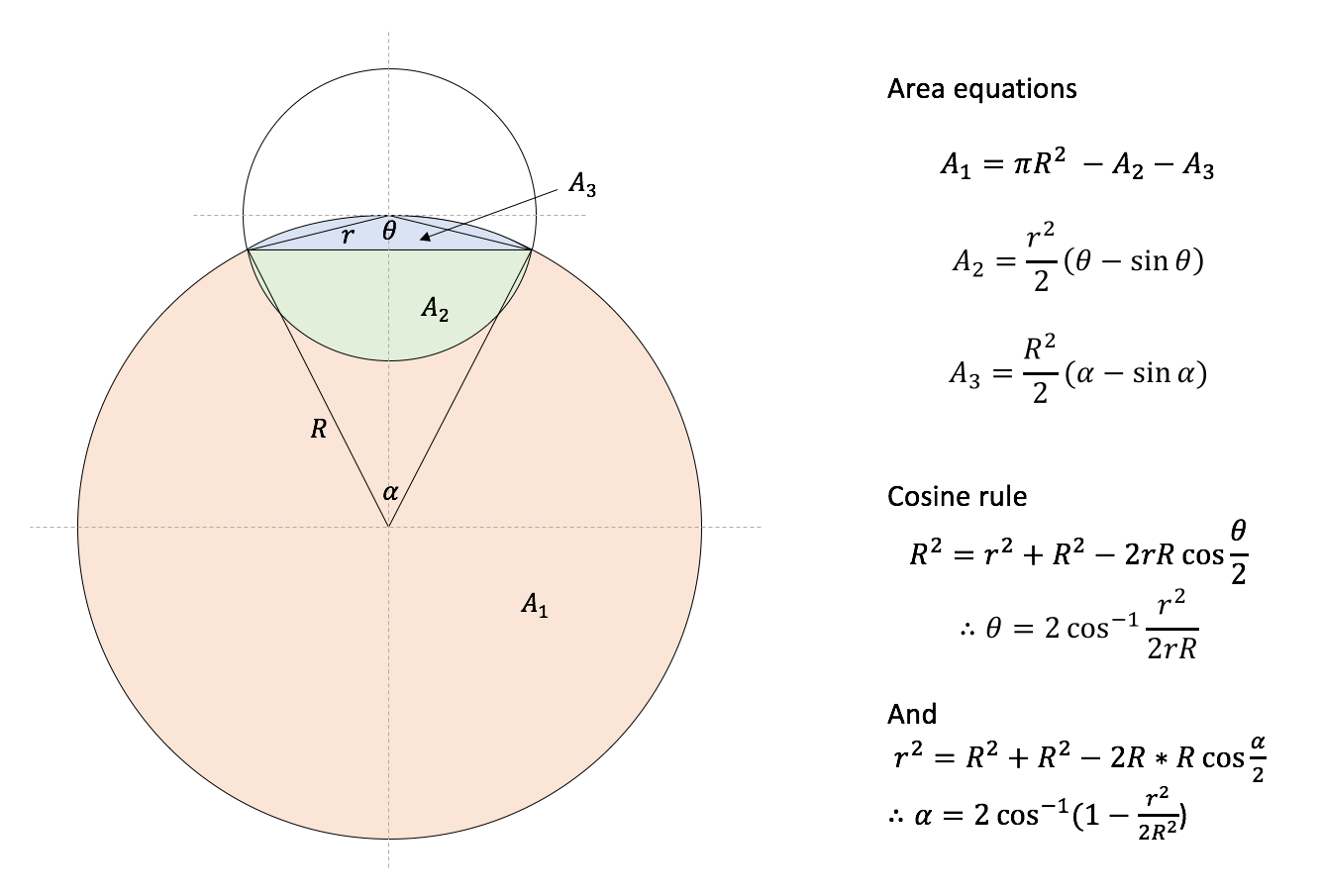


**Figure S2. Phylogenetic distribution of sexual size dimorphism and intromission duration.**

**
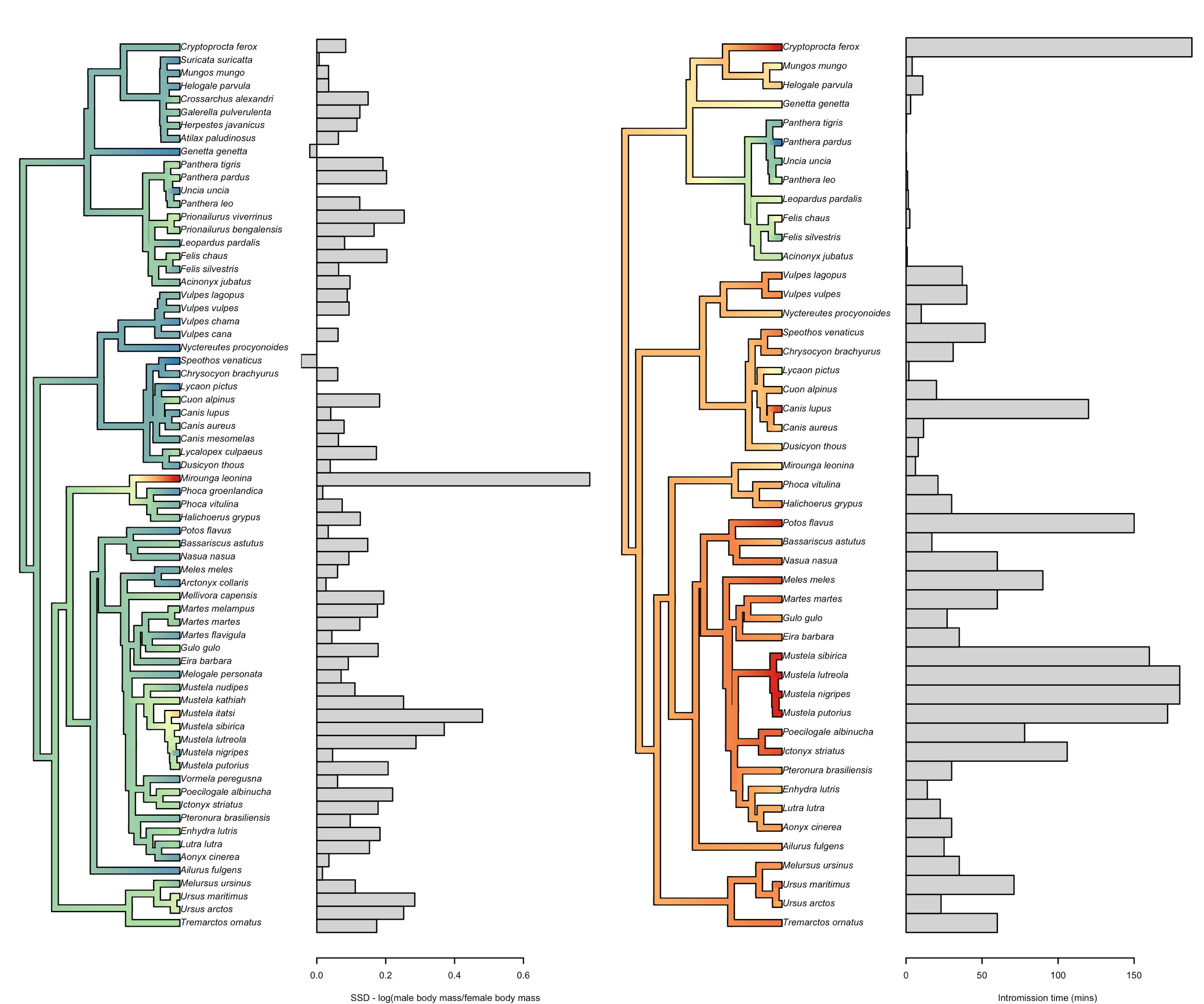
**
