# Supplementary material for: Testing hypotheses for the function of the carnivoran baculum using finite-element analysis
Source: Proc Biol Sci. 2018 Sep 19;285(1887):20181473. doi: 10.1098/rspb.2018.1473 (PMC6170803; doi:10.1098/rspb.2018.1473)
Supplement: MATLAB code for groove calculations [file rspb20181473supp2.docx]

% Testing hypotheses for the function of the Carnivoran baculum using finite element analysis

% Charlotte A. Brassey, James D. Gardiner & Andrew Kitchener 2018

% jdg@liverpool.ac.uk

% Baculum groove model maths

close all

clear all

% Radius and area of non-grooved baculum

R_original = 5;

A_original = pi*R_original^2;

% Groove radii

r = 0.5:0.5:4.5;

% run loop for each groove radius

for r_loop = 1:length(r)

% use optimisation routine (fminbnd) on groove mathematics to calculate the new outer radius

% of grooved baculum so that its cross sectional area matches ungrooved baculum

% ('groove_math' and calc_area_difference' functions are at bottom of script)

[R_new(r_loop)] = fminbnd(@(R) calc_area_difference(R,R_original,r(r_loop)),R_original,2*R_original);

% calculate area of new grooved baculum as double check to ensure it matches ungrooved model

A(r_loop) = groove_maths(R_new(r_loop),r(r_loop));

% calculate area of a range of potential outer radii (these are used for plotting purposes only)

R_loop = linspace(R_original,1.2*R_original);

for i = 1:length(R_loop)

A_loop(r_loop,i) = groove_maths(R_loop(i),r(r_loop));

end

% plot the results

figure('color','w')

subplot(2,1,1)

plot(R_loop,A_loop(r_loop,:),'k')

hold on

h = refline(0,A_original);

set(h,'color','k','linestyle','--')

plot(R_new(r_loop),A,'ro')

box off

xlabel('Radius (mm)')

ylabel('Area (mm^2)')

axis([xlim 50 120])

angle = 0:0.01:2*pi;

circle_x = sin(angle);

circle_y = cos(angle);

subplot(2,1,2)

h(1) = patch(R_original*circle_x,R_original*circle_y,'k');

h(2) = patch(R_new(r_loop)*circle_x+3.5*R_original,R_new(r_loop)*circle_y,'k');

axis equal

h(3) = patch(r(r_loop)*circle_x+3.5*R_original,r(r_loop)*circle_y+R_new(r_loop),'w');

set(h,'linestyle','none')

axis([-9 27 -6 6])

axis off

% write out figures to file

if ~isdir('groove_maths_figures')

mkdir('groove_maths_figures')

end

print('-djpeg',fullfile('groove_maths_figures',['baculum_' num2str(R_new(r_loop)) '_groove_' num2str(r(r_loop)) '.jpeg']))

close all

end

% output results to csv file

% create matrix to ouput

MMM = zeros(length(r)+1,2);

MMM(1,:) = [0 R_original];

MMM(2:end,:) = [r' R_new'];

% write file

fid = fopen('groove_model_dimension.csv','w');

fprintf(fid,'%s\n','Groove radius,Baculum radius');

fclose(fid);

dlmwrite('groove_model_dimension.csv',MMM,'-append')

% function to produce variable (area_difference) that is to be minimised by

% the optimiser 'fminbnd' above (i.e. optimiser tends variable towards zero).

% 'area_difference' is calculated as the difference between cross sectional

% area of original non-grooved baculum and the current grooved baculum.

function area_difference = calc_area_difference(R,R_original,r)

area_difference = abs(pi*R_original^2 - groove_maths(R,r));

end

% function to calculate the cross sectional area of the grooved baculum

% (see paper for figures and equations associated with the maths)

function A1 = groove_maths(R,r)

theta = 2*acos((r^2)/(2*r*R));

alpha = 2*acos(1-(r^2)/(2*(R^2)));

A2 = ((r^2)/2)*(theta - sin(theta));

A3 = ((R^2)/2)*(alpha - sin(alpha));

A1 = pi*R^2 - A2 - A3;

end
